# Back to the Basics of SARS-CoV-2 Biochemistry: Microvascular Occlusive Glycan Bindings Govern Its Morbidities and Inform Therapeutic Responses

**DOI:** 10.3390/v16040647

**Published:** 2024-04-22

**Authors:** David E. Scheim, Peter I. Parry, David J. Rabbolini, Colleen Aldous, Morimasa Yagisawa, Robert Clancy, Thomas J. Borody, Wendy E. Hoy

**Affiliations:** 1 US Public Health Service, Commissioned Corps, Inactive Reserve, Blacksburg, VA 24060, USA; 2 Children’s Health Research Clinical Unit, Faculty of Medicine, The University of Queensland, South Brisbane, QLD 4101, Australia; p.parry1@uq.edu.au; 3 Department of Psychiatry, Flinders University, Bedford Park, SA 5042, Australia; 4 Kolling Institute, Faculty of Medicine and Health, The University of Sydney, St Leonards, NSW 2064, Australia; 5 College of Health Sciences, University of KwaZulu-Natal, Durban 4041, South Africa; aldousc@ukzn.ac.za; 6 Satoshi Omura Memorial Research Institute, Kitasato University, Tokyo 108-8641, Japan; 7 Louis Pasteur Center for Medical Research, Kyoto 606-8225, Japan; 8 Emeritus Professor, School of Medicine and Public Health, University of Newcastle, Newcastle, NE1 7RU, Australia; 9 Centre for Digestive Diseases, Five Dock, NSW 2046, Australia; 10 Emeritus Professor of Medicine, University of Queensland, Herston, QLD 4029, Australia

**Keywords:** SARS-CoV-2, spike protein, COVID-19, sialic acid, hemagglutination, red blood cell, microvascular occlusion, hemagglutinin esterase, pharmacokinetics

## Abstract

Consistent with the biochemistry of coronaviruses as well established over decades, SARS-CoV-2 makes its initial attachment to host cells through the binding of its spike protein (SP) to sialylated glycans (containing the monosaccharide sialic acid) on the cell surface. The virus can then slide over and enter via ACE2. SARS-CoV-2 SP attaches particularly tightly to the trillions of red blood cells (RBCs), platelets and endothelial cells in the human body, each cell very densely coated with sialic acid surface molecules but having no ACE2 or minimal ACE2. These interlaced attachments trigger the blood cell aggregation, microvascular occlusion and vascular damage that underlie the hypoxia, blood clotting and related morbidities of severe COVID-19. Notably, the two human betacoronaviruses that express a sialic acid-cleaving enzyme are benign, while the other three—SARS, SARS-CoV-2 and MERS—are virulent. RBC aggregation experimentally induced in several animal species using an injected polysaccharide caused most of the same morbidities of severe COVID-19. This glycan biochemistry is key to disentangling controversies that have arisen over the efficacy of certain generic COVID-19 treatment agents and the safety of SP-based COVID-19 vaccines. More broadly, disregard for the active physiological role of RBCs yields unreliable or erroneous reporting of pharmacokinetic parameters as routinely obtained for most drugs and other bioactive agents using detection in plasma, with whole-blood levels being up to 30-fold higher. Appreciation of the active role of RBCs can elucidate the microvascular underpinnings of other health conditions, including cardiovascular disease, and therapeutic opportunities to address them.

## 1. Introduction

As continuing breakthroughs in genetics have been prioritized in medical research funding of recent decades, certain biochemical findings of the past have faded from collective memory, including those central to the morbidities of COVID-19 and options for mitigation. In particular, the well-established bindings of the spike protein (SP) of SARS-CoV-2 and other coronaviruses to surface glycans on blood, endothelial and other host cells and their pathogenic consequences have been largely overlooked. With grounding provided in a recent in-depth review [1] and dozens of other works, including [2,3,4,5], key principles of glycan biochemistry essential to understanding and treating COVID-19 and its post-acute sequelae (long COVID) are presented below.

As will be detailed, coronavirus attachment to host cell sialic acid (SA) residues located at the tips of cell surface glycans is an essential and overlooked pathological step. After making its initial attachment to SA, which is ubiquitously distributed on eukaryotic cell surfaces [6,7,8], the virus can then slide over to a host cell receptor for cellular entry, fusion and replication, e.g., via the replication receptor ACE2 for SARS-CoV-2. This is not a new discovery but is rather well-established coronavirus biochemistry known for decades and recently confirmed with multifaceted evidence for SARS-CoV-2 [1]. Ignoring these biochemical fundamentals and assuming that only the replication receptor, ACE2, is of interest for SARS-CoV-2 has led to failures to interpret important pathological sequelae needed to understand clinical symptomatology. These oversights include the following:

1. The central role in SARS-CoV-2 pathology of the 25 trillion red blood cells (RBCs) and close to one trillion each of platelets and endothelial cells in the average human adult has been underappreciated because they have either no ACE2 [9,10,11] or, for endothelial cells, minimal ACE2 [12,13,14,15,16]. Yet all three cells have millions to billions of SA surface molecules per cell (see Appendix A), to which SARS-CoV-2 SP strongly binds, forming attachments that are key to the severe vascular-based morbidities of COVID-19.
2. As an example of the oversight noted above, investigators have struggled to explain clinical observations of the infiltration of endothelial cells by SARS-CoV-2 SP and associated vascular damage under the assumption that ACE2 is the sole host cell attachment point for the virus, given that endothelial cells have minimal ACE2 [17,18] (but they have billions of SA molecules per cell [19]).
3. More broadly, disregard for the active physiological role of RBCs yields unreliable or erroneous reporting of pharmacokinetic parameters being routinely obtained for most drugs and other bioactive agents as detected in plasma, with their whole-blood levels being up to 30-fold higher.

The binding of SARS-CoV-2 SP to host cell glycans, notably for RBCs, platelets and endothelial cells, proceeds within the broader framework of inflammatory and coagulatory pathways that underlie the vascular morbidities of severe COVID-19. These pathways of blood cell aggregation, inflammation and coagulation are intricately intertwined, with, for example, RBC aggregation serving as a trigger for the coagulation cascade and ensuing thrombosis [20,21,22,23]. The biochemical processes and direct consequences of SARS-CoV-2 SP binding to host cell glycans, however, are most clearly considered within a narrower focus, and their importance is illustrated, for example, by the in vivo elicitation of the main severe morbidities of COVID-19 by experimentally induced RBC aggregation in vivo, as described below.

## 2. Coronaviruses including SARS-CoV-2 Attach to Host Cells via Sialylated Glycans

Coronaviruses use a variety of host cell receptors for replication, including DPP4 for MERS, ACE2 for SARS and SARS-CoV-2, and APN for HCoV-229E [24]. Yet more important for the pathology of SARS-CoV-2 and other coronaviruses is the mechanism by which they initially attach to host cells, which is via glycoconjugate molecules (glycans) on the cell surface [1,3,4,6,25,26,27]. Sialic acid (SA), the most common terminal monosaccharide (sugar monomer) for vertebrates [28], is the specific attachment point on host cell glycans for SARS-CoV-2 SP [3,29,30,31]. Following attachment, the virus slides over to its receptor target for fusion and replication. The ratio of one million or more SA molecules to one hundred or fewer ACE2 receptors per typical human cell (see Appendix A) indicates why SA is the most readily available initial attachment point of SARS-CoV-2 to host cells.

### 2.1. RBCs and Platelets Mount a Nonspecific Immune Response by Attaching to Certain Pathogens

After SARS-CoV-2 penetrates the bloodstream through a compromised alveolar–capillary barrier [32,33] and attaches to SA on blood cell surfaces, reciprocally, RBCs, the most abundant cells in the human body [34,35], and platelets mount a primal immune response against such SA-binding pathogens [1,4]. RBCs and platelets (both heavily sialylated, with no ACE2 [1,9,10,11,19,36]) attach to such pathogens, forming virally interlaced RBC aggregates, and deliver them to leukocytes or to macrophages in the liver and spleen for phagocytosis [1,4]. Notably, glycophorin A, a sialoglycoprotein, with one million of its strands extending from each RBC’s surface [37], has no other known physiological role other than for this primal immune response [28,38]. For severe COVID-19 infections, however, the extent of RBC clumps formed can exceed the host’s capacity to sequester them.

### 2.2. Multifaceted Evidence for High-Affinity Bindings of SARS-CoV-2 SP to Host Cell Glycans

High-affinity bindings of SARS-CoV-2 SP to sialylated glycans on host cell surfaces have been repeatedly confirmed in preclinical and clinical studies [1,4]. These studies used nanoparticle arrays of SA derivatives [29] and nuclear magnetic resonance (NMR) spectroscopy [30] and detected SP-induced RBC clumping using the hemagglutination assay [39]. Clinically, SP traces were found on 41% of RBCs from hospitalized COVID-19 patients [40]. When SARS-CoV-2 SP was injected into zebrafish embryos, small RBC aggregates formed, and blood flow slowed within minutes, as thrombosis developed in capillaries, arteries and veins [41]. A competitive SP-binding agent coinjected with SP blocked this thrombogenic effect [41]. Two in vitro studies that failed to detect attachments of SARS-CoV-2 SP to SA [42] or to a sialylated cellular receptor [43] used microarray detection instead of the nanoarray techniques required to support the multivalent bindings that form durable attachments [1,4].

## 3. Clinical Consequences of Glycan Attachment from SARS-CoV-2 SP to Blood and Endothelial Cells

Many studies have found that after COVID-19 gains infectious penetration in the lungs, in severe infections, the SARS-CoV-2 virus penetrates the bloodstream through a compromised alveolar–capillary barrier [32,33]. Severe morbidities, including hypoxia and blood clotting, are then caused by vascular damage and occlusion [1,4,44,45,46]. Indeed, with trillions of RBCs, platelets and endothelial cells per human adult, each heavily sialylated [1,4,19], it is unsurprising that SARS-CoV-2 SP can severely damage the human vasculature. RBC clumps were found in the blood of most of the severe COVID-19 patients in several studies [1,23,47,48,49]. The SARS-CoV-2 virus or SP was frequently detected on endothelial cells in severe COVID-19 patients, with heavily damaged endothelial cells also being observed [1]. Virally induced RBC clumping is reflected in a significantly increased erythrocyte sedimentation rate (ESR) [50,51,52,53,54] and decreased [55] hematocrit levels in severe COVID-19 patients. Also, von Willebrand factor (vWF), a mediator of immunothrombosis associated with endothelial inflammation, was sharply increased in COVID-19 [56,57] and long COVID patients [58] vs. controls (see Appendix B). The potential for RBC aggregation and endothelial damage to trigger the coagulation cascade, resulting in thrombosis, is indicated in [20,22,23] and other studies [1].

### RBC Clumping and Endothelial Damage Cause Microvascular Occlusion, Hypoxia and Blood Clots

**Figure 1:**
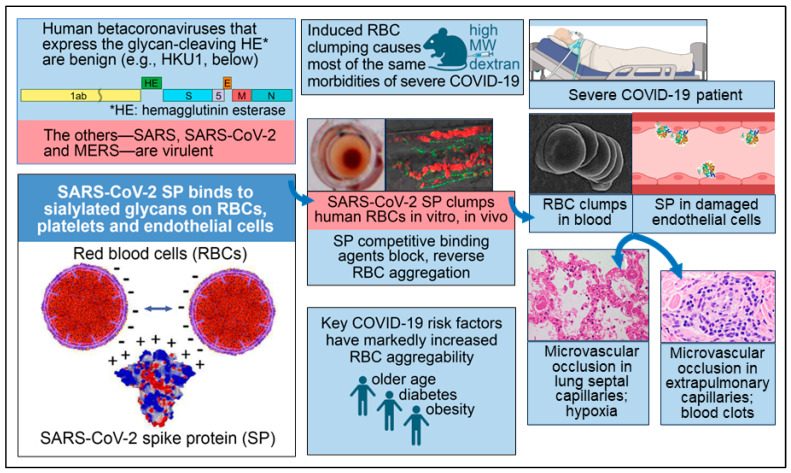
Key facets of the biochemical underpinning of the severe morbidities of COVID-19. Reproduced under CC-BY 4.0 from Scheim et al. 2023 [1]. Lower left panel: SARS-CoV-2 SP, which is positively charged, binds to RBCs, platelets and endothelial cells, the surfaces of which are densely coated with negatively charged sialylated glycans. Upper left panel: the two human betacoronaviruses that express the SA-cleaving enzyme HE are benign, while the other three—SARS, SARS-CoV-2 and MERS—are virulent. Upper center panel: RBC aggregation experimentally induced in vivo by injecting high molecular weight dextran (HMWD) caused most of the same morbidities of severe COVID-19. Middle center panel: An interlaced sheet of RBCs in the hemagglutination assay (**left**) and RBC clumps in zebrafish embryos (**right**) are shown, both induced by the introduction of SARS-CoV-2 SP. Lower center panel: Three key risk factors for COVID-19 morbidity—older age, diabetes and obesity—are each associated with markedly increased RBC aggregation. **Middle right panel**: An electron microscopic image of RBC clumps in blood is shown and SP in damaged endothelial cells is depicted, with both being commonly observed in severe COVID-19 patients. **Lower right panel**: Microvascular occlusion in lung septal capillaries is associated with hypoxia, and microvascular occlusion in extrapulmonary capillaries is associated with blood clots.

Corresponding to the RBC aggregates found in most patients with severe COVID-19 in the studies noted above and associated triggers of the coagulation cascade, many studies have reported microvascular occlusion in both the pulmonary and extrapulmonary vasculature of such patients [1,4]. RBC clumps and microthrombi in the lungs have been considered to be likely causes of hypoxemia, as reflected in decreased peripheral oxygen saturation (SpO2) in severe COVID-19 patients [1,4]. Microthrombi elsewhere in the body, including in the heart, kidneys and liver, were frequently observed in autopsy examinations of COVID-19 patients, often with accompanying multiorgan damage and blood clots [1,4]. The attachment of SARS-CoV-2 SP to host cell glycans underlying these morbidities of severe COVID-19 is diagrammed in Figure 1.

## 4. Substantiation of the Biochemical Underpinnings of Severe COVID-19 Morbidities

The key role of SARS-CoV-2 SP bindings to host cell glycans and associated blood cell aggregation in the severe morbidities of COVID-19 has been substantiated through the following pre-clinical and clinical evidence. For the first three points below, the RBC, the most abundant cell in the human body, was a key focus of most of the related studies, but similar effects were also observed for platelets. The evidence is as follows:

1. RBC aggregation experimentally induced in several animal species by injecting the polysaccharide HMWD caused most of the same morbidities of severe COVID-19, notably microvascular occlusion, hypoxia and blood clots. In studies dating back to the 1940s in dogs, rabbits, mice, hamsters and other animals, RBC clumping was induced within minutes to hours after injection of HMWD, followed by the morbidities noted, with the molecular bridging of RBCs by HMWD being a hypothesized mechanism. Low molecular weight dextran (LMWD) inhibited and reversed this RBC aggregation and microvascular occlusion when RBC clumping had not progressed to clotting [1].
2. Three of the major risk factors for COVID-19 mortality—older age, diabetes and obesity—are each associated with significantly increased RBC aggregation and microvascular occlusion [1,59,60].
3. Three of the generic drugs that have been most closely studied for potential clinical benefits against COVID-19 either reduce RBC aggregation or specifically inhibit virally induced RBC aggregation by competitively binding to SARS-CoV-2 SP [1].
4. For mammalian species, the degree of clinical susceptibility to COVID-19 correlates with the degree of aggregability of RBCs with *p* = 0.033 [1].
5. Of the five human betacoronaviruses, the two that express hemagglutinin esterase (HE), an enzyme that releases viral bindings to host cell sialylated glycans, are benign (the common cold viruses HKU1 and OC43). The other three—SARS, SARS-CoV-2 and MERS—are virulent, even though the viral loads for COVID-19 and the common cold infections are about the same [1].

An important finding related to experimentally induced RBC clumping by HMWD and its inhibition and reversal by LMWD is that RBC aggregation occurs transiently even in healthy mammals under conditions of slow blood flow, with larger clumps sequestered via a distributed network of arterioles and a pulmonary catch–trap architecture [61,62]. When the extent of RBC clumping exceeds the capacity of this sequestration network or when fibrin-stabilized microthrombi are formed, however, that is no longer readily reversible, and the morbidities observed in these studies are manifested.

## 5. SARS-CoV-2 SP Unattached to Virus Induces Microvascular Occlusion

An experimental in vivo system to study the effects of SARS-CoV-2 SP unattached to whole virus in blood was provided by IV injection of an mRNA COVID-19 vaccine, which induces synthesis of SP by host cells, with the trillion endothelial cells lining blood vessels being suitable for this role [1]. In studies in mice [63] and rats [64], the range of adverse effects caused by IV injection of the BNT162b2 vaccine (distinct from the intramuscular (IM) administration route used clinically) included marked blood hypercoagulability along with pericardial damage, electrocardiogram changes and other abnormalities that reflected myocardial injury. Similar myocardial injuries were common adverse effects in the HMWD-induced RBC clumping studies [1]. All of the mice in both the IV- and IM-injected groups of the mouse mRNA injection study had myocardial WBC infiltration and cardiomyocyte degeneration and necrosis vs. no effects in saline-injected controls.

Clinically, a COVID-19 vaccine made from inactivated whole virus, the Sinovac-CoronaVac vaccine, caused no changes to the vascular density (VD) of flowing retinal blood vessels (which excludes occluded, non-flowing vessels), as determined by optical coherence tomography angiography [65,66]. CoronaVac and similar antigen-based vaccines differ from mRNA and adenovector-DNA COVID-19 vaccines in that the latter’s lipid–nanoparticle or adenoviral carrier envelopes transfect cell and tissue membranes far from the injection site and produce unregulated, potentially large amounts of SP for prolonged periods up to months [2].

The Pfizer-BioNTech BNT162b2 mRNA vaccine, however, caused small but statistically significant reductions (*p* < 0.001) in various VD measures [66,67], indicative of microvascular occlusion. At four weeks after vaccination, seven of these VD reductions persisted at statistically significant levels [66]. In another study that used PET/CT scans to track myocardial fluorodeoxyglucose F 18 (FDG) uptake, an indicator of myocardial injury [68,69], that value was abnormally high and significantly greater in mRNA-vaccinated subjects vs. unvaccinated controls, as detected 1–180 days after vaccination (median of 4.8 vs. 3.3, *p* < 0.0001) [70]. Similar potential risks without overt clinical manifestations were indicated from cardiac test markers 2–10 weeks after COVID-19 mRNA vaccinations vs. pre-vaccination values in 566 patients at a cardiac clinic, with an increase in the five-year predicted risk of acute cardiac events from 11% to 25% [71].

The risk period for the occurrence of possible microvascular complications post-COVID-19 vaccination has not been established, but mass spectrometry analysis of whole blood detected SP in 50% of mRNA-vaccinated subjects up to six months after vaccination [72]. Another study found that of 16 COVID-19 mRNA-vaccinated patients hospitalized afterward for myocarditis, all had significant levels of SARS-CoV-2 SP in the blood, while 45 asymptomatic, vaccinated subjects had no detectable SP [73]. Studies which include one recently conducted by a multinational collaboration that examined the health records of 99 million COVID-19 vaccinated individuals [74] and a Yale study of adverse effects manifested after COVID-19 vaccination [75] can help to harmonize post-vaccination signs of microvascular occlusion and myocarditis from retinal VD and myocardial FDG uptake as noted above and from ECG abnormalities [76,77] with safety/toxicity signals corresponding to overt clinical events.

## 6. Rapid Reversal of COVID-19 Hypoxia by Competitive Binding to SARS-CoV-2 SP

Two of the three generic drugs that received the most attention as potential COVID-19 therapeutics, hydroxychloroquine (HCQ) and fluvoxamine, have significant activity in reducing RBC and platelet cell aggregation [1]. A more closely targeted molecular mechanism for mitigating the virulence of SARS-CoV-2 SP by competitive binding is indicated for the third generic drug of major interest, ivermectin (IVM), a macrocyclic lactone that has been dispensed in over four billion human doses worldwide since 1987 [78,79]. IVM had the strongest or close-to-strongest binding affinity to SARS-CoV-2 SP in four in silico studies that collectively screened over 1000 molecules [1]. One molecular modeling study that focused on IVM binding to SARS-CoV-2 SP at its receptor-binding domain (RBD), its region of attachment to host cell ACE2, however, found only low affinity binding [80]. Yet in silico examination of IVM binding to 21 sites distributed across the SP’s RBD and N-terminal domain (NTD) found high-affinity binding to eight of these sites, all but one on the NTD, the SP region which governs its attachments to host cell glycans [81] (see Appendix C). Six other molecular modeling studies confirmed high-affinity bindings of IVM to SARS-CoV-2 SP [1,82].

**Figure 2:**
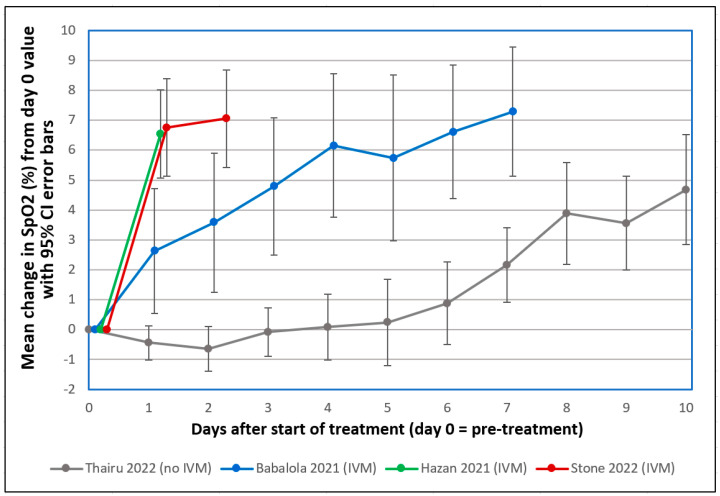
Mean changes in oxygen saturation (SpO2) for severe COVID-19 patients following treatments including or excluding IVM. Reproduced from Stone et al. (2022) [83] (CC-BY 4.0). Graphs show SpO2 values for patients having pre-treatment (day 0) values ≤ 93%. The y-axis value for day n is the mean of changes in SpO2 values from day 0 to day n, with error bars designating 95% confidence intervals. ● Thairu et al. (2022) [84,85]: 26 patients with median age of 45 years treated with different combinations of lopinavir/ritonavir, remdesivir, azithromycin, enoxaparin, zinc sulfate and vitamin C. ● Stone et al. (2022) [83]: 34 patients with median age of 56.5 treated with IVM, doxycycline and zinc. ● Hazan et al. (2021) [86]: 19 patients with median age of 63 treated with IVM, doxycycline and zinc. ● Babalola et al. (2021) [85,87]: 19 patients with median age of 33 treated with IVM, zinc and vitamin C, with some also being given azithromycin and hydroxychloroquine. Patients in the three studies using IVM (Stone, Hazan and Babalola) were all on room air.

Several observations of recovery of COVID-19 patients in severe respiratory distress 1–2 days after IVM treatment, with accompanying sharp increases in SpO2, prompted early interest in this drug in 2020 [1]. This striking SpO2 normalization, as tracked in three clinical studies summarized in Figure 2 and Table 1 below, sometimes occurring within hours after IVM administration [83], paralleled the rapid disaggregation by IVM of RBC clumps that formed after SARS-CoV-2 SP was added to human RBCs in vitro [39]. Sharp increases in SpO2 one day after IVM treatment in these three clinical studies contrasted distinctly, far outside 95% confidence intervals, with a flat SpO2 curve under standard treatment. A similar flat SpO2 curve was tracked in several other clinical studies during the first 1–2 weeks of severe or moderate COVID-19 under standard treatment [1].

The probabilities that the IVM-based treatments yielded greater SpO2 increases than the standard treatment on day 1 or 2 by chance, as listed in Table 1, were very small (*p* < 0.0015) for the Babalola study and infinitesimally small (*p* < 5.5 × 10^−9^) for the other two IVM studies that used a triple therapy of IVM, doxycycline and zinc. A case series that monitored pre- and post-treatment SpO2 values in 71 patients over a 10-day treatment period for COVID-19 who were given the same triple therapy likewise found consistent SpO2 normalization, with means of 93.3% to 98.1% pre- to post-treatment, respectively [89]. Although these results are consistent with the mostly positive results on IVM efficacy from more than 20 randomized controlled trials (RCTs) for COVID-19 treatment conducted in 2020 through mid-2021 [79,90], they may seem incongruous given two such RCTs conducted in 2021 [91] and 2022 [92] that received widespread attention, both of which concluded that IVM provided no statistically significant benefits vs. placebo. Yet both of these RCTs violated core scientific norms, calling their credibility into question (see details [1,93]).

In the 2021 RCT, IVM was substituted for placebo doses for 38 of the 398 total patients, a mistake that was discovered a month later, and blinding was broken by the study’s use of sugar water as the placebo for one-third of the patients (liquid IVM has a bitter taste) [91,94]. Adverse events that are distinctive to the high IVM dose used (transient and non-critical) occurred at almost identical rates in the IVM and placebo arms, while over-the-counter sales of IVM surged in the study region during the study period [94,95].

Despite repeated inquiries, coauthors of the 2022 study (the TOGETHER trial, IVM arm) refused to disclose four of its key outcome numbers, namely, per-protocol deaths and hospitalizations, treatment vs. placebo [93]. These four numbers are of key importance given critiques by the US Food and Drug Administration [96] and National Institutes of Health [97] of the primary outcome used in all arms of that platform trial. Instead, a coauthor of that study directed inquiring scientists to the ICODA data repository, the data source listed in the study’s data sharing statement [93,98]. After two months of futile attempts by scientists to obtain the data from ICODA, however, an ICODA manager disclosed that ICODA never held this study’s data and that she had instructed its authors to stop citing it as their data source [99]. As of 1 April 2024, however, 20 months after notification by the ICODA manager [99] and others [100] to study coauthors that their data were never at ICODA, two TOGETHER trial sister publications with mostly overlapping sets of coauthors with its IVM trial (all having ClinicalTrials.gov registry number NCT04727424) still list their data sharing source as ICODA, which never held their data [101,102].

Based on the infectious characteristics of Omicron variants of SARS-CoV-2 noted in Section 11, which sharply limit penetration into the blood, it is unclear whether the clinical benefits of IVM against pre-Omicron variants as noted above would apply to typically milder Omicron infections. Only modest such benefits in time to recovery were suggested in a platform trial that compared IVM treatment of COVID-19 patients enrolled up through July 2022, an Omicron-dominated period, with controls in a pre-Omicron-dominated period [103]. The study found 1.6% vs. 4.4% rates of hospitalizations and 0.14% vs. 0.37% rates of deaths, IVM vs. controls, with no death rates reported for a concurrent subset of controls and other serious flaws noted [104].

Competitive binding by IVM to SARS-CoV-2 SP appears to underlie the rapid, sharp normalization by IVM of SpO2 values and alleviation of the accompanying respiratory distress in severe pre-Omicron COVID-19 patients. This clinical effect parallels the rapid disaggregation by IVM of RBC clumps that formed after SARS-CoV-2 SP was added to human RBCs in vitro [39]. As noted above, RBC aggregation is readily reversible, as commonly occurs in healthy mammals, with significant masses of RBC clumps nevertheless detrimental to blood oxygenation [1]. In contrast, it does not appear that dissolution of fibrin-enmeshed blood clots, mitigation of endothelial damage or a significant reduction in viral load could occur within one day, even if IVM could cause these effects. However, an anti-inflammatory effect mediated by positive allosteric modulation of the alpha-7 nicotinic receptor by IVM [81] would occur rapidly and might additionally contribute to the noted SpO2 normalization by IVM in that short timeframe.

## 7. Obstacles to the Deployment of Repurposed Generic Drugs

As outlined above, in most COVID-19 research of the past three years, a lack of knowledge or appreciation of how SARS-CoV-2 and other coronaviruses initially attach to host cell surface glycans led to ACE2 being considered the sole host cell receptor of interest for SARS-CoV-2. Also largely overlooked was the associated key role of the heavily sialylated RBCs, platelets and endothelial cells, with trillions of each in the human body, in the key morbidities of severe COVID-19. Under the assumption that since SARS-CoV-2 SP does not replicate, it must be harmless, SP was chosen as the immunogen for most COVID-19 vaccines. Compounding the scientific oversights noted, however, was the vulnerability of medical science to commodification, a subject that has engaged the contributions of some of science’s most distinguished scholars, including current and past editors of leading scientific journals [105,106,107,108,109,110,111,112,113].

Richard Horton, Editor-in-Chief of *The Lancet*, for example, wrote in 2015 that plagued by “flagrant conflicts of interest”, “much of the scientific literature, perhaps half, may simply be untrue” [105]. Financially driven biases in medicine have manifested in the marginalization of some generic drugs in competition with patented offerings. A prime example was a treatment consisting of two antibiotics and bismuth for *H. pylori* (peptic ulcers), a previously intractable condition, which was shown to be 96% curative in a clinical trial conducted by Thomas Borody in 1990 [114]. That triple-therapy cure was rapidly deployed in Australia, preventing an estimated 18,665 deaths up through 2015 [115]. It was not widely used in the rest of the world, however, until the late 1990s, after the patents for two best-selling palliative drugs for that condition expired [116]. The related discovery of the bacterial cause (*H. pylori*) of peptic ulcers was honored with the Nobel Prize for Medicine in 2005. (Borody was a coinvestigator of one of the clinical studies shown in Figure 2 that found rapid, sharp increases in SpO2 after IVM-based triple therapy of severe COVID-19 [86].)

The use of IVM to treat COVID-19 was likewise subjected to questionably based restrictions [117,118,119,120], yet real-world evidence in large populations demonstrated the safety and efficacy of IVM against COVID-19. In Peru, excess deaths decreased 14-fold during four months of mass IVM use in 2020, until a new president elected on November 17 restricted its use, after which excess deaths then increased 13-fold over the next two months [121]. A rigorous state-by-state analysis of IVM use in Peru’s 25 states using national health data that aligned with WHO summary data found a correlation between the extent of IVM use and reductions in excess deaths, by state, with *p* < 0.002 [121].

In Uttar Pradesh, the largest state in India, having a population of 229 million, COVID-19 deaths fell by 97%, from 328 to 10 per day (seven-day moving averages) between May 7 and 7 July 2021, after mass distribution of IVM, doxycycline, zinc with vitamins and acetaminophen tablets began [121]. The cumulative total of COVID-19 deaths per million population in Uttar Pradesh from 7 July 2021 to 1 April 2023 was 4.3, 0.27% of that figure in the US (1596.3) for the same period [121].

## 8. Ignoring RBCs Yields Inaccurate Blood Levels of Drugs and SARS-CoV-2 SP

An RCT for COVID-19 prophylaxis that yielded significant clinical benefits 42 days after a single IVM dose led us to conjecture that RBCs could provide a persisting reservoir for IVM and other drugs (see Appendix D) and that pharmacological values typically determined in plasma or serum could accordingly be inaccurate. A deep literature search finally resolved this pharmacological conundrum. This RBC-binding effect proved not to apply for IVM [122], but it did apply for rapamycin, a widely used drug that is a chemical cousin of IVM, which has a striking, 30-fold ratio of whole-blood-to-plasma concentrations [123,124,125]. This effect also proved to apply to dozens of other drugs [126,127] and bioactive agents [128], with blood-to-plasma ratios greater than 10 for several [126,127,128]. Such binding of drugs and other bioactive agents to RBCs provides extended persistence in circulation [127], with increased opportunity for physiological potency beyond what concentration values in plasma would indicate. Yet this effect has been obscurely reported, pharmacokinetic parameters are still typically detected only in plasma or serum, and erroneous values of significant consequence have been reported for SARS-CoV-2 SP and for another bioactive molecule considered in the next section.

Blood concentrations of SARS-CoV-2 SP are reported in almost every case using plasma or serum, but this glycoprotein binds strongly to RBCs [1], e.g., with SP traces being found on 41% of RBCs from hospitalized COVID-19 patients in one study [40]. Although methodological differences preclude exact comparisons, one mass spectroscopic examination of whole blood found SP in 50% of blood samples from subjects up to six months after mRNA COVID-19 vaccination [72]. Other studies that used plasma or serum, however, found much lower SP levels [73,129,130], e.g., no detectable SP in any subject 10 days post-vaccination [130]. This substantial underassessment of SARS-CoV-2 SP in blood detected using plasma or serum, which may be on the order of or greater than the 30-fold such effect for rapamycin, exemplifies the pitfalls of overlooking the glycan bindings of SP. Moreover, it has confounded assessments of persisting SP in long COVID and post-vaccination syndrome.

## 9. Limiting RBC Aggregation Can Enhance Cardiovascular Health, Cognitive Function and Longevity

As noted above, elevated RBC aggregation and microvascular occlusion are found with older age, diabetes and obesity [1,59,60], and these three are also major risk factors for the largely vascular-based morbidities of severe COVID-19 [1]. These same three risk factors are also associated with significantly increased incidence of cardiovascular disease [131,132,133]. Aside from causing damage associated with microvascular occlusion, RBC aggregation can induce atherosclerosis through mechanisms including increased blood viscosity and forces of traction exerted by the blood on the arterial wall [134,135]. Elevated erythrocyte sedimentation rate (ESR), an indicator of RBC aggregation, was found in multivariate analyses to be closely correlated with cardiac [136] and carotid [137] atherosclerosis and to cardiac mortality, with a closer correlation of cardiac mortality to ESR (*p* < 0.0001) than to elevated cholesterol [136,138]. For the incidence of carotid atherosclerosis, the correlations to ESR and to a direct measure of RBC aggregation in blood were also much higher than its correlation to C-reactive protein (CRP) [137], suggesting that the correlations to indicators of RBC aggregation were not merely reflections of inflammation. Three agents that bind to RBCs, lower RBC aggregation and have shown reductions in the incidence of cardiovascular disease are briefly considered below.

Resveratrol (RSV), a polyphenol component of red wine and other dietary sources, was found in clinical and animal studies to bind to RBCs [139], limit RBC aggregation [140], decrease ESR [141,142] and improve microvascular circulation [143]. In heart failure patients, RSV yielded an improved exercise capacity that was significantly correlated with reduced RBC aggregation [140]. In mice, increased microvascular density and decreased microvascular abnormalities in the brain in the RSV vs. control group were paralleled by enhanced performance in a maze task [143]. Human RSV levels after oral intake were 3.2 times higher in blood vs. plasma [144] due to its RBC binding, but its underassessed plasma concentrations were erroneously cited in several reports as indicative of a disparity between peak RSV blood levels and two- or three-fold higher levels required for beneficial effects in vitro [145,146,147].

Chloroquine and its analog, hydroxychloroquine (HCQ), also have blood-to-plasma concentration ratios greater than 3.0 [126,148], and for these drugs as well, this effect was associated with decreased RBC and platelet aggregation, reductions in microvascular occlusion and associated physiological benefits found clinically and in vivo [149,150,151]. Three RCTs that studied HCQ use for rheumatoid arthritis and lupus found significant reductions in cardiovascular events and morbidity vs. non-HCQ controls [152,153,154].

RSV, however, is most compellingly positioned for evaluation as a practical intervention to reduce the risks of cardiovascular disease in light of the real-world evidence associated with the “French paradox”. Mortality from coronary heart disease (CHD) in France in recent decades was found to be one-half to one-third of that in other countries, including the US, UK and Sweden, despite higher French consumption of saturated fat [155]. RSV in red wine, a mainstay of the French diet, has been proposed as the key to that country’s decreased CHD mortality [155]. Ethanol at concentrations commensurate with moderate alcohol consumption, however, has also been found to decrease aggregation of RBCs [156], with the same aggregation-limiting effect observed for platelets [157]. Thus, the ethanol in red wine might also contribute to the lower French CHD mortality, yet multivariate analyses of the effects of light-to-moderate ethanol consumption show either no reduction in CHD mortality [158] or reductions of 12–20% [159], much less than those noted above for France.

## 10. Discussion

This article summarizes and extends more detailed works by its coauthors and others, including [2,3,4,5], which delve into complexities not considered here. It bears reaffirmation that the binding of SARS-CoV-2 SP to RBCs, platelets and endothelial cells which underlies the blood cell aggregation, vascular damage and related severe morbidities of COVID-19 proceeds within a broader framework of cascading inflammatory and coagulatory pathways. At the same time, however, there is stark simplicity to the disregard of well-established biochemistry—including the initial attachment of SARS-CoV-2 and other coronaviruses to host cell surface glycans, and the primal immune defense by which RBCs and platelets clump and sequester such viruses—that has characterized most COVID-19 research. Probing the pathology of SARS-CoV-2 through the nearly exclusive lens of its replication and its replication receptor, ACE2, has resulted in the noted significant oversights concerning opportunities and risks of COVID-19 therapeutics.

Although additional cellular receptors, including neuropilin-1 and the alpha-7 nicotinic acetylcholine receptor, are likely targets of SARS-CoV-2 for certain cell types [1,160], SA is of prime interest because it serves as the initial attachment point on all host cells for this and other coronaviruses, as noted, and is ubiquitous on eukaryotic cell surfaces [6,7,8]. Although the attachment of SARS-CoV-2 SP to sialylated host cell glycans has been demonstrated in multiple studies, a complete understanding of how different chemical bonds support such attachment remains to be achieved. Most conspicuous is the attraction between the positively charged SARS-CoV-2 SP [39,161,162,163] and the negatively charged, densely distributed SA on the surfaces of RBCs, platelets and endothelial cells, as depicted in the bottom left panel of Figure 1. (The associated electrostatic repulsion between blood and endothelial cells is key to smooth blood flow [19].) Covalent glycosidic bonds from SA to other sugar monomers may also join glycans populating the 22 N-glycosylation sites and the four O-glycosylation sites of SARS-CoV-2 with interlocking glycans on host cells [1,30,164,165,166,167]. 

Although this review focuses on attachment to host cells by SARS-CoV-2 SP in the context of that for other coronaviruses, SA is also the attachment point for pathogens of other viral families [168,169,170]. For influenza, the virus’s membrane fusion glycoprotein, hemagglutinin, is complemented by its SA-cleaving enzyme, neuraminidase [3,168,170,171], which serves a role analogous to hemagglutinin esterase (HE) for the two benign human betacoronaviruses, the common cold viruses HKU1 and OC43. 

## 11. Conclusions

The well-established glycan biochemistry of coronaviruses, as multiply confirmed for SARS-CoV-2 SP, reveals how this viral SP attaches to the densely sialylated surfaces of RBCs, platelets and endothelial cells and to other host cells, causing RBC aggregation, pulmonary and extrapulmonary microvascular occlusion, hypoxia and blood clots. Especially vulnerable to these severe morbidities are patients of older age, diabetes or obesity, who have significantly increased baseline levels of RBC aggregation. These basic principles of SARS-CoV-2 biochemistry and the associated key roles of the trillions of RBCs, platelets and endothelial cells in the average human adult, which have no ACE2 or (for endothelial cells) minimal ACE2 but very dense SA surface coatings, however, have been largely marginalized in most COVID-19 research of the past three years. 

This disregard of glycan biochemistry has resulted in the following significant oversights concerning SARS-CoV-2 SP. Blood levels of SP, as with almost all drugs and other bioactive agents, are commonly misdetermined using plasma, an invalid detection method for RBC-binding molecules, which can have whole-blood-to-plasma ratios as high as 30 to one. Indeed, SP levels in the blood, which persist to detectable levels months after mRNA COVID-19 vaccination [72], have only been accurately determined using whole blood (see Section 8). A second oversight was the assumption that SARS-CoV-2 SP is harmless because it cannot replicate, yet it binds strongly to RBCs and endothelial cells, induces RBC aggregation in vitro [39] and causes damaging related effects in vivo [63,64].

The use of SARS-CoV-2 SP as the immunogen for most COVID-19 vaccines raises concerns for the current versions that use Omicron subvariants, since SP from one Omicron lineage was found to have ten times the hemagglutinating activity of SP from prior variants [39]. Corresponding multi-fold increases in the net positive charge of the SP of Omicron vs. prior variants [39,161,162,163] account for this markedly increased attachment strength to the negatively charged RBC surface. Although Omicron infections are milder than those of prior variants, as related to its less efficient replication in the lung alveolar epithelium [172,173], which is the virus’s portal into the blood [32], the much greater hemagglutinating activity of Omicron’s SP increases its potential risks for use in vaccinations. Therefore, there may be additional risks associated with the new generation of Omicron-based COVID-19 vaccines, which had no human testing [174] and, as noted in an NIH media advisory of 19 July 2022 [175], have reduced efficacy vs. those based on prior variants [176,177,178].

The RBC-disaggregating effect of IVM caused by its competitive binding to SARS-CoV-2 SP, which underlies its striking clinical benefits for pre-Omicron variants (see Section 6), would likely not apply for Omicron infections due to the Omicron properties noted above. Yet IVM may prove useful for the treatment of long COVID, in which SP has been found to persist in blood [179,180,181,182], and for prevention of COVID-19 [183,184,185,186]. Given the clear record of safety of IVM in four billion human doses worldwide [78,79], with this safety record specifically noted by the Nobel Prize committee in 2015 in honoring the discovery of this drug [187], the availability of IVM to treat or prevent COVID-19 and long COVID will provide far more benefits than risks to public health.

Expanding beyond the scope of COVID-19, gaps in knowledge of glycan biochemistry and the associated role of RBCs have extended to underappreciation of the role of RBC aggregation in cardiovascular disease. For RSV, for example, which may be key to the major reductions in CHD mortality in France vs. other countries in past decades, these oversights extend even to errors in its basic pharmacology, with three-fold underassessed values for RSV concentrations reported using plasma vs. whole blood. As noted above, RSV has been found in clinical and animal studies to bind to RBCs, limit RBC aggregation, and improve microvascular circulation, exercise capacity and cognitive function.

The scope of health impacts from RBC aggregation and microvascular occlusion is exemplified by the finding that subjects of ages 56–75 had ten-fold the percentage of occluded microvessels in the bulbar conjunctiva as for those of ages 16–35 (30% vs. 3%) [59], with ocular microvascular occlusion having been found to mirror such conditions elsewhere in the body [188,189]. Moreover, elevated ESR, an indicator of RBC aggregation, was found to be highly correlated to cardiovascular mortality (*p* < 0.0001), with a stronger correlation than that to elevated cholesterol. It is thus conceivable that a public health program to monitor ESR levels and restrict them within normal limits using RSV or related agents might yield equal or greater benefits than current strategies of monitoring and normalizing cholesterol levels. In a best-case scenario, biochemical insights gleaned through research that was prompted by the COVID-19 pandemic may result in enduring benefits for public health.

## Figures and Tables

**Table 1:** Mean changes in oxygen saturation (SpO2) for severe COVID-19 patients one or two days following treatments including or excluding IVM, with statistical significance of compared values.

Clinical Series	Used IVM	Day 1 after Start of Treatment			Day 2 after Start of Treatment		
		No.*	Mean ± SE of ∆SpO2 ^†^	*p*-Value ^‡^	No.*	Mean ± SE of ∆SpO2 ^†^	*p*-Value ^‡^
Stone et al. 2022 [83]	Y	33	6.76 ± 0.80	9.75 × 10^−10^	34	7.06 ± 0.80	2.84 × 10^−10^
Hazan et al. 2021 [86]	Y	19	6.55 ± 0.70	5.45 × 10^−9^	-	-	-
Babalola et al. 2021 [87]	Y	19	2.63 ± 0.99	0.00149	19	3.58 ± 1.11	0.0001901
Thairu et al. 2022 [84]	N	26	-0.42 ± 0.27	-	26	-0.62 ± 0.35	-

* The number of patients having SpO2 values on the day noted. ^†^ SE is standard error; ∆SpO2 is the change in SpO2 from day 0 (pre-treatment) to the designated day (1 or 2). ^‡^ The *p*-value shown is the probability of the set of SpO2 changes for study patients being stochastically equivalent to those for Thairu et al. (2022), as calculated using the Mann–Whitney U test (two-tailed) [88]. The underlying data on SpO2 changes for individual patients in these four studies are shown in Tables S1–S5.

## Data Availability

Not applicable.

## Supplementary Materials

The following are available online at https://www.mdpi.com/article/10.3390/v16040647/s1, Table S1: Changes in SpO2 for 34 COVID-19 patients treated with IVM, doxycycline and zinc, as reported by Stone et al. (2022), Table S2: Changes in SpO2 for 19 COVID-19 patients treated with IVM, doxycycline and zinc, as reported by Hazan et al. (2021), Table S3: Changes in SpO2 for 19 COVID-19 patients treated with IVM, zinc and vitamin C, with some also given azithromycin and hydroxychloroquine, as reported by Babalola et al. (2021), Table S4: Changes in SpO2 for 26 COVID-19 patients treated without IVM using different combinations of lopinavir/ritonavir, remdesivir, azithromycin, enoxaparin, zinc sulfate and vitamin C, as reported by Thairu et al., Table S5: Means and standard errors of SpO2 changes from day 0 to day 1 and from day 0 to day 2 for the full set of 26 patients from Thairu et al. (2022) and for the subset of 18 patients who were on room air (without oxygen supplementation or ventilation).

## Abbreviations

The following abbreviations are used in this manuscript:

CHD	coronary heart disease
COVID-19	coronavirus disease 2019
CRP	C-reactive protein
ECG	electrocardiogram
ESR	erythrocyte sedimentation rate
FDG	fluorodeoxyglucose F 18
HCQ	hydroxychloroquine
HE	hemagglutinin esterase
HMWD	high molecular weight dextran
IM	Intramuscular
IV	Intravenous
IVM	Ivermectin
LMWD	low molecular weight dextran
long COVID	post-acute sequelae of COVID-19 (or PASC)
NMR	nuclear magnetic resonance
NTD	N-terminal domain
RBC	red blood cell
RBD	receptor-binding domain
RCT	randomized controlled trial
RSV	resveratrol
SA	sialic acid
SARS-CoV-2	severe acute respiratory syndrome coronavirus 2
SP	spike protein
SpO2	peripheral oxygen saturation
VD	vascular density
vWF	Von Willebrand factor

## Appendix A. Notes and Calculations on Surface Area and Extent of Sialylation of RBCs and Endothelial Cells in Human Vasculature

In an average human adult, the blood contains 25 trillion RBCs [34,35], each with a surface area of 163 µm^2^ [126], yielding a total surface area of about 4075 m^2^. The blood also contains nearly one trillion platelets [190], and the vasculature is lined with one trillion endothelial cells having a total surface area of about 1000 m^2^ [191]. RBCs, platelets and endothelial cells are each heavily sialylated, with about 35 million SA monosaccharides per RBC [19,192], a more than ten-fold greater surface density of SA on platelets and endothelial cells than on RBCs [19,36], and about 25–50 billion SA molecules (vs. 175 ACE2 receptors) per endothelial cell [12,19] (using the figure of 1000 µm^2^ for the approximate surface area of an endothelial cell [191]). The surface of one type of immortalized human glomerular endothelial cell was found to consist of 50% SA [193]. RBCs [9] and platelets [10,11] have no ACE2, while endothelial cells have minimal ACE2 [12,13,14,15,16]. Whereas mammalian cells of all major tissues typically contain at least one million SA molecules per cell [8], ACE2 is either absent or expressed at counts of one hundred or less per cell in most human cell types [12,194].

## Appendix B. Significantly Increased Erythrocyte Sedimentation Rate (ESR), Decreased Hematocrit Level and Increased von Willebrand Factor (vWF) in Severe COVID-19 Patients and Long COVID Patients

Significantly increased blood values for erythrocyte sedimentation rate (ESR) and decreased hematocrit levels were found in severe COVID-19 patients. ESR was increased [50,51,52,53,54], and hematocrit level decreased [55] in COVID-19 patients with greater disease severity, with *p* < 0.001 for both. This ESR increase is consistent with the RBC clumping induced by SARS-CoV-2 SP, while the decreased hematocrit level could result from larger RBC clumps being sequestered, as it occurs for such clumps that form transiently even in healthy mammals, via a distributed network of arterioles and a pulmonary catch–trap architecture [61,62]. Note that RBC aggregation, which is readily reversible, is different from the formation of fibrin-enmeshed blood clots. Also, von Willebrand factor (vWF), an indicator of endothelial inflammation, was sharply increased in severe vs. non-severe COVID-19 patients [195,196] and in COVID-19 [56,57] and long COVID patients [58] vs. controls.

## Appendix C. High-Affinity Binding of IVM to SARS-CoV-2 SP at the SP’s NTD

Aminpour et al. (2022) found by in silico computations that IVM binds with high affinity (<−7.0 kcal/mol) to seven sialoside binding sites or other glycan-binding sites on SARS-CoV-2 S1, six on the N-terminal domain (NTD) and one on the receptor-binding domain (RBD), for the RBD in the open (“up”) position [81]. For the RBD in the closed (“down”) position, these binding energy values of <−7.0 kcal/mol were obtained for two additional glycan binding sites (eight total) on the NTD and the same one on the RBD. As a measure of the significance of that binding energy value, binding energies of <−7.0 kcal/mol predicted efficacy for a large set of HIV inhibitors with 98% sensitivity and 95% specificity in another study [197].

## Appendix D. A Persistent Physiological Effect of IVM 42 Days after a Single Standard Dose, Despite a Plasma Half-Life of 72 h for the Major Metabolites of IVM

An RCT for COVID-19 prevention administered just one dose of IVM at 12 mg (about 150 μg/kg, at the low end of a standard dose) to 617 subjects on day one of a 42-day observation period [184]. Three other agents were each administered daily to subjects in other study arms over that same period. IVM at that single low dose on day one yielded the best results of the four agents, with statistically significant reductions close to 50% in both symptomatic COVID-19 patients (*p* = 0.01) and patients with acute respiratory symptoms (*p* = 0.0034) vs. controls on day 42.

A significant physiological effect of IVM 42 days after oral administration, however, appears puzzling given its elimination half-life. IVM is absorbed into the blood and distributed in body tissues to peak levels typically within 4–8 h after oral dosage, with an average elimination half-life of IVM determined in plasma of roughly 18 h [198,199,200,201]. This plasma half-life is four-fold greater, three days, however, for the major metabolites of IVM [198], but that still does not appear consistent with a significant physiological effect 42 days after a single dose. If IVM were to bind with moderate or high affinity to RBCs, that could provide a reservoir for the drug that would not be detected from its plasma levels, yet IVM was found, in fact, to bind minimally to RBCs [122], although it is conceivable that its active metabolites bind more strongly to RBCs. Other studies indicate persistence of IVM in tissue and persistent physiological effects in humans [198,200,202] and rabbits [203], respectively, from many days to one month after a single dose.
