# Supplementary material for: Back to the Basics of SARS-CoV-2 Biochemistry: Microvascular Occlusive Glycan Bindings Govern Its Morbidities and Inform Therapeutic Responses
Source: Viruses. 2024 Apr 22;16(4):647. doi: 10.3390/v16040647 (PMC11054389; doi:10.3390/v16040647)
Supplement: Supplementary file 1 [file viruses-16-00647-s001.zip › Scheim et al. 2024. Back to the Basics, Supplementary Tables.pdf]

Back to the Basics of SARS-CoV-2 Biochemistry: Microvascular Occlusive Glycan Bindings Govern its Morbidities and Inform Therapeutic Responses

Supplementary Tables

Table S1. Changes in SpO2 for 34 COVID-19 patients treated with IVM, doxycycline and zinc, as reported by Stone et al. (2022) [1]. For the elapsed times of x=12, 24, and 48 hours after first IVM dose, the SpO2 value shown for a given patient is that at the latest post-treatment time ≤ x. All pre-treatment SpO2 values are from within one hour before treatment. All patients were on room air (without oxygen supplementation) and all had pretreatment SpO2 ≤ 93%.

Age	Sex	SpO2 (%)			
		Pre-treatment	at +12 hours	at +24 hours	at +48 hours
25	M	79	92	93	93
32	F	88	95	95	95
35	M	87	95	95	95
37	M	92	99	99	98
38	F	76	88	89	91
40	F	93	-	99	99
42	M	90	90	90	93
44	F	89	93	93	93
44	M	87	93	93	94
45	M	83	96	95	95
49	F	66	90	90	90
50	F	89	93	93	93
50	M	92	-	95	95
52	F	90	93	93	93
55	F	82	-	-	91
55	M	81	-	88	92
56	F	87	93	94	94
57	F	84	-	89	88
58	M	85	94	94	94
58	M	89	95	95	95
59	M	79	82	85	85
59	M	92	93	93	93
61	F	87	-	98	99
61	F	92	-	96	96
62	M	88	89	93	91
62	M	91	94	94	94
66	M	85	84	84	84
66	M	88	94	94	94
68	M	89	95	95	95
68	M	90	-	96	96
71	F	89	-	94	95
75	M	80	92	90	90
80	F	85	91	91	91
≥90	M	88	95	98	98

Table S2. Changes in SpO2 for 19 COVID-19 patients treated with IVM, doxycycline and zinc as reported by Hazan et al. (2021) [2]. The data shown are for the 19 of 24 patients who had SpO2 values recorded within 24 hours after first IVM dose, and whose pre-treatment SpO2 was ≤ 93%; this excludes one patient with a pre-treatment SpO2 value of 97%; the rest actually all have pretreatment SpO2 values ≤ 90%). All patients were on room air (without oxygen supplementation).

Age	Sex	SpO2 (%)	
		Pre-treatment	within +24 hours
66	M	90.0	94.0
62	M	77.0	87.0
75	M	88.0	96.0
66	F	89.0	95.0
43	F	88.0	94.0
62	M	86.5	91.0
57	M	88.0	96.0
94	F	88.0	94.0
63	F	90.0	96.0
47	M	84.0	91.0
69	F	88.0	91.0
69	M	88.0	91.0
46	F	87.0	94.0
86	M	88.0	95.0
59	F	90.0	95.0
92	M	85.0	91.0
63	M	90.0	96.0
57	M	73.0	90.0
87	M	90.0	95.0

Table S3. Changes in SpO2 for 19 COVID-19 patients treated with IVM, zinc and vitamin C, with some also given azithromycin and hydroxychloroquine, as reported by Babalola et al. (2021) [3,4]. The data shown are for the 19 patients with pre-treatment SpO2 values ≤ 93% and who were on room air (without oxygen supplementation).

		SpO2 (%)		
Age	Sex	Pre-treatment	at +1 day	at +2 days
29	M	93	92	93
34	M	92	94	94
60	F	92	94	97
34	m	90	90	90
33	F	78	90	97
43	M	92	94	96
32	M	92	90	94
23	M	90	94	94
19	F	90	92	92
21	F	90	90	92
48	M	89	99	88
33	F	92	92	92
24	M	92	92	94
32	F	92	92	92
32	M	90	90	94
89	M	91	92	94
56	M	92	96	97
41	F	92	93	94
32	M	76	89	89

Table S4. Changes in SpO2 for 26 COVID-19 patients treated without IVM, using different combinations of lopinavir/ritonavir, remdesivir, azithromycin, enoxaparin, zinc sulfate and vitamin C, as reported by Thairu et al. (2022) [4,5]. The data shown are for the 26 patients with pre-treatment SpO2 values ≤ 93%. Those (7 patients) who had oxygen supplementation are highlighted in yellow, and one on ventilation is highlighted in pink. The other 18 were on room air.

Age	Sex	SpO2 (%) Pre-treatment	at +1 day	at +2 days
45	F	88	88	88
35	F	88	88	87
63	F	81	81	82
47	M	91	88	85
52	M	91	91	91
41	F	88	88	84
45	F	87	87	87
54	M	88	88	88
36	F	88	87	88
54	F	84	84	83
54	M	88	88	88
38	F	87	88	88
55	M	84	84	84
32	M	82	83	82
58	M	88	88	87
58	M	88	88	88
44	M	88	88	88
32	M	88	88	88
25	M	88	88	88
49	M	86	88	88
26	M	88	85	88
59	M	88	87	87
45	F	88	88	89
39	M	84	84	84
45	M	88	85	85
35	M	88	84	84

Table S5. Means and standard errors of spo2 changes from day 0 to day 1 and from day 0 to day 2 for the full set of 26 patients from Thairu et al. (2022) and for the subset of 18 patients who were on room air (without oxygen supplementation or ventilation).

		Change in SpO2 (%) Day 0 to Day 1	Day 0 to Day 2
All 26 patients	Mean	-0.423	-0.615
	Standard Error	0.267	0.347
The 18 patients on room air	Mean	-0.500	-0.556
	Standard Error	0.381	0.459

References

1. Stone, J.C.; Ndarukwa, P.; Scheim, D.E.; Dancis, B.M.; Dancis, J.; Gill, M.G.; Aldous, C. Changes in SpO2 on Room Air for 34 Severe COVID-19 Patients after Ivermectin-Based Combination Treatment: 62% Normalization within 24 Hours. *Biologics* **2022**, *2*, 196-210.
2. Hazan, S.; Dave, S.; Gunaratne, A.W.; Dolai, S.; Clancy, R.L.; McCullough, P.A.; Borody, T.J. Effectiveness of ivermectin-based multidrug therapy in severely hypoxic, ambulatory COVID-19 patients. *Future Microbiology* **2022**, *17*, 339-350.
3. Babalola, O.E.; Ndanusa, Y.; Adesuyi, A.; Ogedengbe, O.J.; Thairu, Y.; Ogu, O. A Randomized Controlled Trial of Ivermectin Monotherapy Versus HCQ, IVM, and AZ Combination Therapy in Covid-19 Patients in Nigeria. *J Infect Dis Epidemiol* **2021**, *7*, 233.
4. Babalola, O.E.; (Bingham University, New Karu, Nigeria). Personal communications, 28 February 2022, 26 January, 2024.
5. Thairu, Y.; Babalola, O.E.; Ajayi, A.A.; Ndanusa, Y.; Ogedengbe, J.O.; Omede, O. A comparison of Ivermectin and Non Ivermectin based regimen for covid 19 in Abuja: effects on virus clearance, Days-to-Discharge and Mortality. *Journal of Pharmaceutical Research International* **2022**, *34*, 1-19.
